# Growth Rate of *Plasmodium falciparum*: Analysis of Parasite Growth Data from Malaria Volunteer Infection Studies

**DOI:** 10.1093/infdis/jiz557

**Published:** 2019-11-04

**Authors:** Leesa F. Wockner, Isabell Hoffmann, Lachlan Webb, Benjamin Mordmüller, Sean C. Murphy, James G. Kublin, Peter O’Rourke, James S. McCarthy, Louise Marquart

**Affiliations:** 1 QIMR Berghofer Medical Research Institute, 4006 Brisbane, QLD, Australia; 2 Institute of Tropical Medicine, University of Tübingen, 72074, Tübingen, Germany; 3 Departments of Laboratory Medicine and Microbiology, University of Washington, Seattle, WA, 98109, USA; 4 Fred Hutchinson Cancer Research Center, Seattle, WA, 98109, USA; 5 School of Medicine, The University of Queensland, 4072 Brisbane, QLD, Australia; 6 Q-Pharm Pty Ltd, 4006 Brisbane, QLD, Australia

**Keywords:** Parasite growth rate, *Plasmodium falciparum* 3D7, statistical models, induced blood stage malaria, volunteer infection studies, CHMI

## Abstract

**Background:**

Growth rate of malaria parasites in the blood of infected subjects is an important measure of efficacy of drugs and vaccines.

**Methods:**

We used log-linear and sine-wave models to estimate the parasite growth rate of the 3D7 strain of *Plasmodium falciparum* using data from 177 subjects from 14 induced blood stage malaria (IBSM) studies conducted at QIMR Berghofer. We estimated parasite multiplication rate per 48 hour (PMR_48_), PMR per life-cycle (PMR_LC_), and parasite life-cycle duration. We compared these parameters to those from studies conducted elsewhere with infections induced by IBSM (n=66), sporozoites via mosquito bite (n=336) or injection (n=51).

**Results:**

The parasite growth rate of 3D7 in QIMR Berghofer studies was 0.75/day (95% CI: 0.73–0.77/day), PMR_48_ was 31.9 (95% CI: 28.7–35.4), PMR_LC_ was 16.4 (95% CI: 15.1–17.8) and parasite life-cycle was 38.8 hour (95% CI: 38.3–39.2 hour). These parameters were similar to estimates from IBSM studies elsewhere (0.71/day, 95% CI: 0.67–0.75/day; PMR_48_ 26.6, 95% CI: 22.2–31.8), but significantly higher (*P* < 0.001) than in sporozoite studies (0.47/day, 95% CI: 0.43–0.50/day; PMR_48_ 8.6, 95% CI: 7.3–10.1).

**Conclusions:**

Parasite growth rates were similar across different IBSM studies and higher than infections induced by sporozoite.

## Background

The growth rate of *Plasmodium* parasites in the blood of infected individuals is a major determinant of parasite biomass and the pathology of malaria [1]. The therapeutic goal of preventing or treating malaria is to control parasite replication, using vaccines or antimalarial chemotherapy. Therefore, the parasite growth rate is an important outcome of malaria clinical trials designed to evaluate an effect on parasite replication after a vaccine-induced antibody response. Furthermore, the parasite growth rate is a key parameter of pharmacometric models used to predict the efficacy of antimalarial drugs [2].

The parasite multiplication rate (PMR) is the fold-change in number of parasites over a life-cycle. The PMR is derived from the log_10_-based parasite growth rate, and is typically expressed as growth across a 48 hour (h) period (PMR_48_), the generally accepted duration of the *P. falciparum* life-cycle. Analysis of historical studies of malaria therapy for syphilis, where parasitemia was determined by microscopy, estimated a PMR_48_ of 8 for several *P. falciparum* strains [3]. PMR_48_ estimates of clinical isolates of *P. falciparum* collected from patients with malaria have varied from 2.3 to 6.0 in ex vivo cultures [4]. However, the effect of adaptation to culture is a key determinant of this variability. PMR_48_ has also been estimated using parasitemia data from volunteer infection studies (VIS) – otherwise known as Controlled Human Malaria Infection (CHMI) studies – conducted to evaluate efficacy of blood stage vaccines. In VIS, healthy subjects are infected by bites of *Plasmodium* -infected mosquitoes [5, 6], by parenteral injection of cryopreserved *P. falciparum* sporozoites [7, 8], or by intravenous injection of *Plasmodium* -infected erythrocytes using the induced blood stage malaria (IBSM) model [9, 10]. The PMR_48_ of 3D7 or NF54, the common *P. falciparum* strains used in VIS, has been reported to range from 7.5 to 14.4 in mosquito bite studies [11–13] and from 10 [14] to 21 [15] in the IBSM model.

The PMR_48_ may differ between malaria-naive individuals and individuals previously exposed to malaria [16], as well as between different parasite strains. The method used to measure parasitemia [17] and the statistical model used to estimate parasite growth rate [18, 19] can also substantially affect PMR_48_estimates. Estimating parasite growth rate accurately is important when developing blood stage vaccines and antimalarial drugs. Shorter parasite life-cycles than the generally accepted 48 h have been estimated by visual interpretation of *P. falciparum* 3D7 parasitemia data in mosquito bite sporozoite studies [17]. However, the duration of *P. falciparum* 3D7 life-cycle in the IBSM model has not been estimated using a statistical model. Accurate estimation of the parasite life-cycle in the IBSM model would allow estimation of PMR per life-cycle (PMR_LC_).

In this study, we analyzed data from IBSM studies conducted at QIMR Berghofer (QIMR-B) in which subjects were inoculated with *P. falciparum* 3D7 under similar experimental conditions [20–32] and parasitemia quantitated by a validated quantitative PCR (qPCR) assay [33]. We estimated the parasite growth rate and parasite life-cycle of *P. falciparum* 3D7, to then calculate PMR_48_ and PMR_LC_. We compared these estimates with our estimates using data from IBSM studies conducted by other research groups [14, 15, 34–37], from mosquito bite sporozoite studies [17, 19, 34, 38], and from cryopreserved sporozoite studies [8, 10, 39–42].

## METHODS

### IBSM Studies from QIMR Berghofer

We analyzed data from 177 malaria-naïve healthy subjects who participated in 14 IBSM studies across 27 cohorts between 2012 and 2017 at Q-Pharm Pty Ltd (Supplementary Table 1). All studies were approved by the QIMR-B human research ethics committee and all subjects provided informed consent (Supplementary Table 1).

Table 1 summarizes characteristics of the QIMR-B IBSM studies analyzed. Subjects were inoculated intravenously on Day 0 with human erythrocytes infected with approximately 1800, 2300, or 2800 viable *P. falciparum* 3D7 parasites. Subjects were treated with an antimalarial drug on Day 7, 8, or 9.

**Table 1:** Summary Details of QIMR-B IBSM Studies

Characteristic	n (%)^a^
**Gender**	
Male	129 (73%)
Female	48 (27%)
**Age**	
18–24	96 (54%)
25–29	50 (28%)
≥30	31 (18%)
**Inoculum Dose (Approximate No. of Viable Parasites)**	
1800	122 (69%)
2300	9 (5%)
2800	46 (26%)
**Treatment Day**	
Day 7	67 (38%)
Day 8	109 (62%)
Day 9	1 (0.6%)
**Ethnicity**	
Caucasian	155 (88%)
Other	22 (12%)

a Number of subjects in each category. Total number of subjects, n=177.

### Parasite Growth Monitoring and Data Processing of IBSM Studies from QIMR-B

Parasite growth was monitored using a qPCR assay targeting the *P. falciparum* 18S rRNA gene using a TaqMan probe [33]. Parasitemia was monitored twice daily after subjects were qPCR-positive until time of antimalarial drug administration. All samples from a subject were analyzed in duplicate or triplicate in a single assay at the end of study. Replicates were geometrically averaged on the log_10_ scale. The limit of detection of the qPOthCR assay was 64 parasites/mL [33]. However, the qPCR assay frequently detected parasite densities below this value; the measured parasite densities were used in the analysis. If one parasitemia replicate was not detected, and the other replicate was positive, the replicate non-detected value was set to 1 parasite/mL to give zero on the log scale and the geometric mean of the positive replicate values and 1 was taken. Non-detected sample values across all replicates before the first positive qPCR measurement were excluded from analysis. However, if parasitemia had been detected by qPCR at previous time points, non-detected parasitemia values were set to 32 parasites/mL (half the limit of detection of the qPCR assay). Other approaches to substitute non-detected values have been reported including substitution methods [12] and modeling techniques to handle censored observations [19, 43]. Processed individual parasitemia data for the 177 subjects are presented in Supplementary Table 2.

### IBSM and Sporozoite Studies from Other Research Groups

We analyzed parasitemia data from IBSM and sporozoite studies conducted by other research groups. These studies used a range of methodologies, including different means of infection (IBSM, mosquito bite, or cryopreserved sporozoites), different *P. falciparum* strains (3D7 or NF54), and different PCR methods to estimate parasitemia: TaqMan qPCR, SYBR Green qPCR, quantitative reverse transcription PCR (qRT-PCR), or nested PCR with fluorescence quantification of band intensity (Table 2). The methodology used to process parasitemia data is summarized in Supplementary Table 3 and Supplementary Methods.

**Table 2:** Summary of Characteristics of IBSM and Sporozoite Studies from Other Research Groups Analyzed in this Report

Study	*P. falciparum*	Inoculum Size^a^	Detection Methodology^b^	Molecular Gene
	Strain	(~ No. of Viable Parasites)		Target
**IBSM**				
QIMR-B [20-32]	3D7	1800 (n=122) 2300 (n=9) 2800 (n=46)	TaqMan qPCR^*^	18S rRNA
Payne et al. (2016) [14]	3D7	690	TaqMan qPCR^*^	18S rRNA
Bijker et al. (2013)^c^ [34]	3D7	1962	TaqMan qPCR^*^	18S rRNA
Duncan et al. (2011) [35]	3D7	250	TaqMan qPCR^*^	18S rRNA
Sanderson et al. (2008) [15]	3D7	1800	SYBR Green qPCR	18S rRNA
Lawrence et al. (2000)^d^ [36]	3D7	Group 1: 141 (n=8) Group 2: 114 (n=9)	Nested PCR and fluorescence quantification of band intensity	STEVOR
Cheng et al. (1997) [37]	3D7	Subject 2: 3000 Subject 3: 3000 Subject 4: 6000 Subject 5: 300	Nested PCR and fluorescence quantification of band intensity	STEVOR
**Sporozoite Mosquito Bite**				
Reuling et al. (2018) [38]	3D7	5 *Anopheles stephensi* mosquitoes	TaqMan qPCR	18S rRNA
Douglas et al. (2013) [17]	3D7	5 *Anopheles stephensi* mosquitoes (combines data from 4 studies)	TaqMan qPCR	18S rRNA
Douglas et al. (2013) [17]	3D7	5 *Anopheles stephensi* mosquitoes (combines data from 9 studies)	SYBR Green qPCR	18S rRNA
Bijker et al. (2013) [34]	3D7	5 *Anopheles stephensi* mosquitoes	TaqMan qPCR	18S rRNA
Coffeng et al. (2017)[19]	NF54	4–7 *Anopheles stephensi* mosquitoes (n=20) 5 *Anopheles stephensi* mosquitoes (n=36) (combines data from 9 studies)	TaqMan qPCR^*^	18S rRNA
**Cryopreserved Sporozoites**				
Sheehy et al. (2013)^e^[8]	NF54	2500 sporozoites intradermally (n=5) 2500 sporozoites intramuscularly (n=3) 25000 sporozoites intramuscularly (n=6)	TaqMan qPCR^*^	18S rRNA
Mordm¨ller et al. (2017) [10]	NF54	3200 sporozoites	qRT-PCR^**^	18S rRNA
Sulyok et al. (2017) [40]	NF54	3200 sporozoites	qRT-PCR^*^	18S rRNA
Murphy et al. (2018) [39]	NF54	3200 sporozoites	qRT-PCR^*^	18S rRNA
MALACHITE [41]	NF54	3200 sporozoites	qRT-PCR^*^	18S rRNA
PREMIVER [42]	NF54	3200 sporozoites	qRT-PCR^*^	18S rRNA

a All inocula used for IBSM trials were produced with *P. falciparum* 3D7 parasites from QIMR-B master cell bank.

b Studies that used a *Plasmodium* 18S rRNA PCR-based methodology that had been analytically validated and compared in an EQA program are marked with an asterisk (*) and were included in the meta-analysis. The study from Mordm¨ller et al (**) was not included in the EQA program but was included in the meta-analysis because the PCR methodology was comparable to those include in the EQA program.

c Only control subjects were included in the analysis.

d Authors of this study divided subjects in two groups for management purposes.

e Four of the 18 subjects reported in the original publication did not get infected with malaria and were not included in the analysis.

Abbreviations: qPCR, quantitative PCR; rRNA, ribosomal RNA; STEVOR, subtelomeric variable open readng frame; EQA, external quality assessment.

### Statistical Models

Pre-treatment parasitemia data from QIMR-B IBSM studies were used to fit log-linear and sine-wave growth models. Data were fitted overall by simultaneously analyzing data from the 177 subjects. Data were also fitted by subject (177 subjects individually) and by cohort (27 cohorts individually). Data from IBSM and sporozoite studies conducted by other research groups were only analyzed overall for all data presented in each of the original publications. Model selection for the random effects structure in mixed-effects models was assessed using the Bayesian Information Criterion and the stability of the parameter estimates.

*Log-linear parasite growth model.* The log-linear model used to estimate parasite growth was:

$${\text{log}}_{10}\left(Y\right)=a+m\times time,$$

where *Y* = parasitemia (parasites/mL) measured by qPCR; *a* = y-intercept, *m* = parasite growth rate per day; *time* = days from inoculation, ranging from first positive PCR timepoint to treatment. The model was fitted by subject using simple log-linear regression, and by cohort and overall using a linear mixed-effects model with a random effect for a estimated using maximum likelihood. The models fitted by cohort assumed that the random effect for a was independent for each subject. For the model fitted overall, a nested random effect for a was included to capture the variability at cohort and subjects-within-cohort levels.

*Sine-wave parasite growth model*. The sine-wave model used to estimate parasite growth was:

$${\text{log}}_{10}\left(Y\right)=a+m\times time+c\times \text{sin}\left(\left(\frac{2\times \pi }{period}\right)\times time+k\right),$$

where *Y, a, m*, and *time* are as above, and *c* = sine-wave amplitude; *period* = duration of the parasite life-cycle in days; *k* = sine-wave phase shift. This model was fitted by subject using non-linear regression, and by cohort and overall using a non-linear mixed-effects model. The same random effects described in the log-linear model were applied. Additionally, as each subject within a cohort received inoculum from the same batch, but the inoculum was not synchronized between cohorts, the model fitted overall included a random effect for the sine-wave phase shift modeled at the cohort level and assumed to be independent of the random effect for *a*.

Model convergence and parameter estimation for the sine-wave models fitted by subject were sensitive to the starting values of the model, which were chosen as the estimated parasite growth parameters of the sine-wave model fitted by cohort, for the cohort the subject belonged to.

For all models, the time variable was centered by its corresponding mean to aid model convergence, calculated either at the overall or cohort levels as per the respective analysis group (calculated at cohort level for subject level analysis).

## PMR estimation

PMR_48_ was estimated as follows:

$${PMR}_{48}=10^{\left(2m\right),}$$

where *m* is the parasite growth rate per day estimated by the log-linear or sine-wave growth models, and 2 days is the accepted parasite life-cycle of 48 h.

PMR_LC_ was estimated as follows:

$${PMR}_{LC}=10^{\left(period\times m\right)},$$

where *m* is the parasite growth rate per day and *period* is the duration of the parasite life-cycle in days, both estimated by the sine-wave model.

### Effect of Gender, Age, and Inoculum Size on Parasite Growth Parameters

The log-linear and sine-wave growth models described above were fitted to data from QIMRB studies stratified by subject gender and age, and by inoculum size (Supplementary Table 1). The inoculum size of 2300 viable parasites was excluded for analysis because of its small sample size (n = 9) (Table 1). The same random effects described above for the log-linear and sine-wave models were applied.

### Statistical Analysis

Parasite growth models were fitted using the package nlme, version 3.1 [44] within R statistical software [45], version 3.3.0. Summary statistics (mean, maximum, minimum, standard deviation) were determined using R. Standard errors were extracted from the appropriate mixed-effects model, and subsequently used to estimate 95% confidence intervals (CI) based on the standard normal distribution. Parasite growth rate estimates are presented as increase in parasitemia per day in log_10_ scale. Parasite life-cycles were estimated per day and transformed as per hour with corresponding 95% CI.

A paired t-test was used to compare parasite growth rates estimated by log-linear and sine-wave models fit by cohort and by subject. Two sample t-tests were used to compare parasite growth rate, sine-wave amplitude, and parasite life-cycle estimated by growth models stratified by gender, age, and inoculum size.

Parasite growth rate and parasite life-cycle of pooled IBSM and sporozoite studies were estimated with random effects meta-analyses using the DerSimonian–Laird estimate [46]. Meta-analysis was performed using package meta, version 4.8-1 [47] within R. Differences in pooled estimates between groups were assessed using the between subgroup heterogeneity Q statistic. Studies that used a *Plasmodium* 18S rRNA PCR-based methodology that had been analytically validated and compared in an external quality assessment (EQA) program [48] were included in the pooled analysis (Table 2). The study from Mordm¨ller et al [10] was also included in the pooled analysis because the PCR methodology was comparable to those compared in the EQA program. All hypotheses were tested at the 5% significance level.

For QIMR-B studies, sensitivity analyses were performed to evaluate the effect of substituting non-detected parasitemia values. Non-detected parasitemia values were substituted by three different values: 1 parasite/mL, 32 parasites/mL (half of the limit of detection of the qPCR assay), and as a missing value.

## RESULTS

### Parasite Growth Rates of QIMR-B IBSM Studies

**Figure 1:**
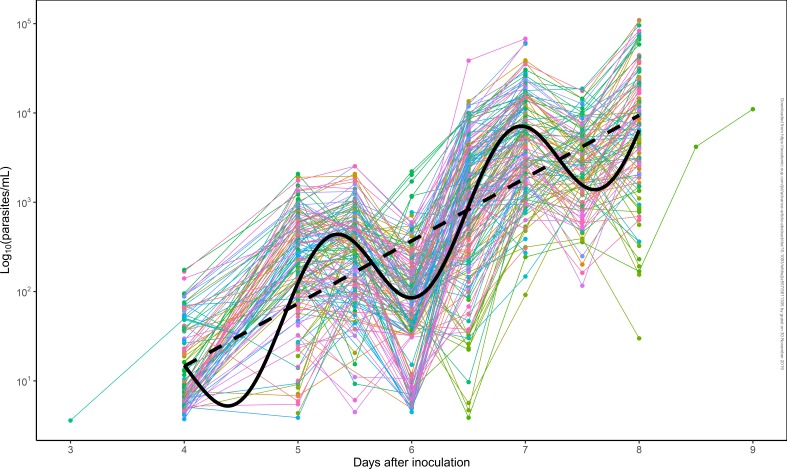
**Parasitemia Data of QIMR-Sudies and Models Fits.** Observed parasitemia data from 177 subjects from QIMR-B studies along with the fixed effects fits of the overall log-linear (black dashed line) and sine-wave models (black continuous line).

Figure 1 shows the individual parasitemia profile of the 177 QIMR-B subjects and the overall fitted log-linear and sine-wave models of parasite growth. The log_10_ parasite growth rate estimated fitting QIMR-B IBSM parasitemia data overall using a sine-wave model was 0.75/day (95% CI: 0.73–0.77/day), the amplitude was estimated as 0.63 log_10_ parasites (95% CI: 0.59–0.66 log_10_ parasites) and the parasite life-cycle was estimated as 38.8 h (95% CI: 38.3–39.2 h). This corresponds to a PMR_48_ of 31.9 (95% CI: 28.7–35.4), and a PMR_LC_ of 16.4. (95% CI: 15.1–17.8) (Table 3).

**Table 3:** Parasite Growth Parameters For IBSM and Sporozoite Studies from Other Research Groups Fitted Overall Using the Log-Linear or Sine-Wave Model

Study	n^a^	**Log-Linear Model**			**Sine-Wave Model**				
		Parasite Growth Rate per Day (95%CI)	PMR_48_ (95%CI)	PMR_LC_ (95%CI)	Parasite Growth Rate per Day (95%CI)	PMR_48_ (95%CI)	PMR_LC_ (95%CI)	Parasite Life-Cycle (h) (95%CI)	Sine-Wave Amplitude (95%CI)
**IBSM**									
QIMR-B [20–32]	177	0.71	25.7	13.8	0.75	31.9	16.4	38.8	0.63
		(0.67–0.74)	(22.2–29.8)	(12.2–15.5)	(0.73–0.77)	(28.7–35.4)	(15.1–17.8)	(38.3–39.2)	(0.59–0.66)
Payne et al. (2016) [14]	27	0.66	20.9	12.9	0.69	24.5	14.7	40.4	0.54
		(0.61–0.71)	(16.8–25.9)	(10.7–15.5)	(0.67–0.72)	(21.5–28.0)	(13.2–16.5)	(39.6–41.2)	(0.48–0.60)
Bijker et al. (2013) [34]	5	0.61	16.7	10.6	0.79	37.3	20.8	40.3	0.56
		(0.46–0.77)	(8.2–34.0)	(5.8–19.3)	(0.67–0.90)	(21.8–63.9)	(13.3–32.7)	(37.1–43.4)	(0.41–0.70)
Duncan et al. (2011) [35]	8	0.69	24.3	19.5	0.73	28.8	22.8	44.7	0.43
		(0.58–0.80)	(14.7–40.3)	(12.2–31.2)	(0.66–0.80)	(21.1–39.3)	(17.1–30.5)	(42.0–47.4)	(0.34–0.53)
Sanderson et al. (2008) [15]	5	0.67	22.2	13.4	0.71	26.0	15.4	40.2	0.73
		(0.50–0.84)	(10.2–48.4)	(7.0–25.8)	(0.58–0.84)	(14.3–47.6)	(9.3–25.4)	(37.6–42.9)	(0.45–1.01)
Lawrence et al. (2000) [36]	17	0.61	16.6	11.5	0.67	22.3	14.9	41.7	0.53
		(0.52–0.69)	(11.0–24.2)	(8.0–15.9)	(0.60–0.74)	(16.2–30.8)	(11.2–19.7)	(39.7–43.8)	(0.42–0.65)
Cheng et al. (1997) [37]	4	0.40	6.3	4.0	0.40	6.3	4.0	36.3	0.18
		(0.35–0.46)	(5.0–8.2)	(3.4–4.9)	(0.35–0.45)	(5.1–8.0)	(3.4–4.8)	(33.4–39.1)	(0.03–0.34)
**Sporozoite**									
**Mosquito Bite**									
Reuling et al. (2018) [38]	16	0.45	7.8	5.6	0.44	7.7	5.5	40.1	0.33
		(0.39–0.51)	(5.9–10.3)	(4.4–7.0)	(0.39–0.49)	(6.0–9.7)	(4.5–6.7)	(38.3–41.9)	(0.23–0.43)
Douglas et al. (2013) - TaqMan [17]	94	0.46	8.3	5.9	0.47	8.7	6.2	40.3	0.37
		(0.44–0.48)	(7.4–9.2)	(5.4–6.5)	(0.45–0.49)	(8.0–9.5)	(5.7–6.6)	(39.7–40.9)	(0.33–0.41)
Douglas et al. (2013) - SYBR Green [17]	165	0.39	5.9	4.3	0.39	6.0	4.4	39.6	0.30
		(0.36–0.41)	(5.2–6.7)	(3.9–4.8)	(0.36–0.42)	(5.3–6.8)	(4.0–4.9)	(38.5–40.8)	(0.24–0.36)
Bijker et al. (2013) [34]	5	0.56	13.2	7.8	0.59	14.8	8.6	38.3	0.44
		(0.42–0.70)	(7.1–24.8)	(4.8–13.0)	(0.43–0.74)	(7.1–30.9)	(4.8–15.4)	(36.8–39.8)	(0.34–0.54)
Coffeng et al. (2017) [19]	56	0.51	10.3	7.7	0.51	10.7	7.9	41.8	0.43
		(0.46–0.55)	(8.2–12.8)	(6.4–9.4)	(0.46–0.57)	(8.4–13.6)	(6.4–9.7)	(40.5–43.2)	(0.37–0.48)
**Cryopreserved**									
**Sporozoites**									
Sheehy et al. (2013) [8]	14	0.62	17.7	11.2	0.67	22.3	13.7	40.4	0.66
		(0.55–0.70)	(12.3–25.5)	(8.3–15.3)	(0.62–0.73)	(17.4–28.7)	(11.1–16.9)	(39.2–41.6)	(0.56–0.77)
Mordmüller et al. (2017) [10]	16	0.54	12.2	6.3	0.51	10.6	5.7	35.3	0.29
		(0.48–0.61)	(9.1–16.4)	(5.1–7.8)	(0.45–0.57)	(8.0–14.1)	(4.6–7.0)	(32.8–37.7)	(0.16–0.41)
Sulyok et al. (2017)^c^ [40]	4	0.58	14.2	NA	NA	NA	NA	NA	NA
		(0.42–0.73)	(7.0–28.4)						
Murphy et al. (2018)^b^ [39]	4	0.51	10.7	NA	NA	NA	NA	NA	NA
		(0.33–0.70)	(4.5–25.3)						
MALACHITE [41]	9	0.49	9.5	4.9	0.52	10.8	5.4	34.0	0.39
		(0.36–0.61)	(5.4–16.8)	(3.3–7.4)	(0.41–0.62)	(6.7–17.4)	(3.8–7.6)	(31.7–36.3)	(0.17–0.60)
PREMIVER [42]	4	0.53	11.5	9.6	0.51	10.7	8.9	44.3	0.47
		(0.42–0.64)	(6.9–19.3)	(5.9–15.4)	(0.44–0.59)	(7.6–15.1)	(6.5–12.3)	(41.7–47.0)	(0.33–0.62)

a Number of subjects included in analysis for each study.

b Sparse data with no cyclic pattern observed. Non-linear models did not result in appropriate fits, so results are excluded.

From the 1128 parasitemia timepoints for the QIMR-B IBSM studies, 26 (2.3%) had non-detected values for all replicates after the first positive value, and 105 (9.3%) had one replicate with non-detected values. Sensitivity of the model to substituted parasitemia values for these non-detected samples and replicates is presented in supplementary material (Supplementary Table 4 and Supplementary Material).

Parasite growth and shape parameters estimated overall were similar to parameters obtained by fitting the data by subject and by cohort (Supplementary Tables 5–7). The mean parasite growth rates estimated using log-linear models were significantly different to parasite growth rates estimated using sine-wave models when fitted by subject (*P* < 0.001) and by cohort (*P* = 0.007). This difference was not significant when only subjects treated on Day 7 were included in the analysis by cohort (Supplementary Table 8).

Analysis of data stratified by gender, age and inoculum size is presented in Supplementary Table 9. The mean parasite growth rate and amplitude in female subjects were significantly higher than in male subjects when using a sine-wave model (*P* < 0.001), but not when using a log-linear model (*P* = 0.10). Subject age did not significantly affect parasite growth or shape parameters. The parasite life-cycle of the 2800 viable parasites inoculum was marginally longer than that of the 1800 viable parasites inoculum when using a sine-wave model (*P* = 0.033), but the opposite pattern was found for amplitude (P = 0.025).

### Comparison of Parasite Growth Parameters of QIMR-B IBSM Studies with IBSM and Sporozoite Studies Conducted by Other Research Groups

Parasite growth and shape parameters estimated using log-linear and sine-wave models fitted overall for data from IBSM and sporozoite studies conducted by other research groups are presented in Table 3.

The parasite growth rate of *P. falciparum* 3D7 estimated using parasitemia data from QIMRB studies was similar to the parasite growth rate estimated using parasitemia data from pooled IBSM studies conducted by other groups (0.75/day [95% CI: 0.73–0.77/day] vs 0.71/day [95% CI: 0.67–0.75/day], *P* = 0.087) (Table 4). The duration of the *P. falciparum* 3D7 life-cycle for all IBSM studies (QIMR-B and studies from other groups) was similar to the *P. falciparum* 3D7 life-cycle from pooled sporozoite mosquito bite studies (40.6 h [95% CI: 38.9–42.3 h] vs 39.7 h [95% CI: 38.4–40.9 h], *P* =0.40). However, the *P. falciparum* 3D7 growth rate from pooled IBSM studies from QIMR-B and other groups (0.73/day, 95% CI: 0.69–0 77/day; PMR_48_ 28.9, 95% CI: 24.1–34.8) was significantly higher (*P* < 0.001) than the *P. falciparum* 3D7 growth rate from pooled sporozoite mosquito bite studies (0.47/day, 95% CI: 0.43–0.50/day; PMR_48_ 8.6, 95% CI: 7.3–10.1).

**Table 4:** Parasite Growth Parameters of Pooled Studies using IBSM and Sporozoite Models Calculated Using Random Effects Meta-Analysis for Sine-Wave Model

IBSM (Datasets Analyzed)^a^	Parasite Growth Rate per Day (95% CI)	PMR_48_ (95% CI)	PMR_LC_ (95% CI)^b^	Parasite Life-Cycle (h) (95% CI)
QIMR-B IBSM (14 studies, 177 subjects)^c^	0.75 (0.73–0.77)	31.9 (28.7–35.4)	16.4 (15.1–17.8)	38.8 (38.3–39.2)
Others IBSM (3 studies, 40 subjects)	0.71 (0.67–0.75)	26.6 (22.2–31.8)	17.2 (14.7–20.1)	41.6 (38.9–44.3)
QIMR-B + others IBSM (17 studies, 217 subjects)	0.73 (0.69–0.77)	28.9 (24.1–34.8)	17.2 (14.7–20.1)	40.6 (38.9–42.3)
**Sporozoites – Mosquito Bite (Datasets Analyzed)**				
3D7 (3 studies, 115 subjects)	0.47 (0.43–0.50)	8.6 (7.3–10.1)	5.9 (5.2–6.8)	39.7 (38.4–40.9)
NF54 (1 study 56 subjects)	0.51 (0.46–0.57)	10.7 (8.4–13.6)	7.9 (6.4–9.7)	41.8 (40.5–43.2)
**Sporozoites – Cryopreserved (Datasets Analyzed)**				
NF54 (4 studies, 43 subjects)	0.56 (0.47–0.65)	13.1 (8.5–20.0)	7.9 (5.6–11.1)	38.5 (34.4–42.6)

a Studies that used a *Plasmodium* 18S rRNA PCR-based methodology that had been analytically validated and compared in an EQA program were included in the meta-analysis (see Table 2): IBSM: Payne et al [14], Bijker et al [34], and Duncan et al [35] studies; sporozoite mosquito bite 3D7: Reuling et al [38], Douglas et al [17], and Bijker et al [34] studies; sporozoite mosquito bite NF54: Coffeng et al 2017 [19]; sporozoites cryopreserved NF54: Sheehy et al [8], Mordm¨ller et al [10], MALACHITE [41], PREMIVER [42].

b Calculated based on the parasite life-cycle estimated from the meta-analysis presented in the last column.

c QIMR-B IBSM studies were treated as one group in the meta-analysis. The overall result from the 14 studies was used. All other studies were treated as individual studies.

Abbreviations: IBSM, induced blood stage malaria; QIMR-B, QIMR Berghofer; EQA, external quality assessment.

The parasite growth rate of *P. falciparum* NF54 estimated using data from sporozoite studies by mosquito bite (0.51/day, 95% CI: 0.46–0.57/day; PMR_48_ 10.7, 95% CI: 8.4–13.6) was comparable (*P* = 0.14) with that of *P. falciparum* 3D7 (0.47/day, 95% CI: 0.43–0.50/day; PMR_48_ 8.6, 95% CI: 7.3–10.1) (Table 4), and comparable (p=0.42) to the sporozoites studies using cryopreserved NF54 sporozoites (0.56/day, 95% CI: 0.47–0.65/day; PMR_48_ 13.1, 95% CI: 8.5–20.0).

## DISCUSSION

The dataset analysed here offers an unique opportunity to characterize the growth of *P. falciparum* in malaria-naïve subjects undergoing experimental infections. We estimated parasite growth rate, PMR_48_, PMR_LC_, amplitude and life-cycle of *P. falciparum* 3D7 by modeling data from 177 subjects of 14 IBSM studies conducted by QIMR-B at a single site using similar conditions. The parasite growth rates estimated for QIMR-B IBSM studies were similar to rates estimated using the same statistical models on data from other IBSM studies that used equivalent molecular methods, thus confirming the robustness of the estimates.

Our results suggest the parasite growth rate is similar in studies using similar means of infection. Our estimates of PMR_48_ in subjects from the studies undertaken by Payne et al [14] and Duncan et al [35] were substantially higher than the estimates reported in their original publications (PMR_48_ ~10 [14], and ~17 [35] respectively), where the intercept was fixed and data were fitted by subject. However, fixing the intercept to the inoculum size by extrapolating beyond the range of measured parasitemia timepoints introduces a confounding effect on estimation of parasite growth rate [18]. Our estimate of the PMR_LC_ in subjects from the report by Coffeng et al [19], where subjects were infected with *P. falciparum* NF54, was marginally lower than the estimates in the original publication, which used a more complex model and Bayesian fitting framework; this suggests using the sine-wave model to estimate parasite growth rate resulted in similar output.

The sine wave amplitude is a good indicator of the synchronicity of the infection, and values were generally higher in IBSM studies than in sporozoite studies, a factor which likely reflects some variation in time of rupture of infected liver schizonts.

The duration of the parasite life-cycle of the 3D7 strain of *P. falciparum* was similar in IBSM and sporozoite studies, and in all cases was shorter than 48 h. The life-cycle estimated for QIMR-B IBSM studies was 38.8 h, whereas in a pooled analysis of IBSM studies from QIMR-B and other research groups the parasite life-cycle was 40.6 h. To our knowledge, this is the first time the parasite life-cycle of *P. falciparum* 3D7 has been estimated for the IBSM model using a sine-wave model. Previously, life-cycle has been fixed to 48 h [3] when sine-wave models were used to estimate parasite growth rate. However, our results suggest that allowing the model to estimate the parasite life-cycle would result in more accurate estimates of parasite growth rate and the derived PMR_LC_.

PMR_LC_ provides an estimation of the average number of parasite progeny produced by a single infected erythrocyte over one replication cycle. According to our meta-analysis, each *P. falciparum* 3D7 parasite infects an average of 17.2 (95% CI: 14.7–20.1) erythrocytes in each life-cycle during IBSM infection. Previous in vitro studies with cultured *P. falciparum* 3D7 have reported the average number of merozoites within a schizont to be 22 [49]; however, the number of merozoites that successfully infected erythrocytes, which is estimated by the PMR_LC_, was not reported.

Lower parasite growth rates in subjects infected by mosquito bite compared with those infected by IBSM have been previously reported [15]. This difference could be due to several reasons. Inocula used to infect subjects in all the IBSM studies were prepared from a single donor unit collected from a volunteer experimentally infected by mosquito bite from a single *P. falciparum* 3D7 in vitro culture. In contrast, parasites derived from mosquitoes used in sporozoite studies are from separate preparations of mosquitoes fed on in vitro cultured parasites. It is possible that there are genetic or epigenetic differences between different parasite lines used for mosquito bite studies. Spence et al [50] reported that the parasite growth rate of *P. chabaudi* in mice was higher when the infection was induced by IBSM than by mosquito bite, and proposed that epigenetic reprogramming during sexual recombination in the mosquito may explain this difference. Furthermore, in mosquito bite studies, typically undertaken to test pre-erythrocytic vaccine efficacy, parasitemia is generally monitored only until infection is confirmed (one to three parasite cycles), whereas in IBSM studies parasitemia is typically measured for four to five parasite life-cycles before treatment. It is possible that the preceding liver stage of sporozoite studies more strongly initiates innate or adaptive immunity that serves to slow subsequent growth in blood stage. Estimates of parasite growth rate will be more accurate as the duration of infection increases, as more data above the limit of detection are available for analysis. In the IBSM studies reported here, all inocula were effectively identical and prepared as the product of a single mosquito infection.

The parasite growth rates estimated using log-linear models appeared to be sensitive to the phase of the growth cycle of the last observation included in analyses. Although the log-linear model is simpler to implement because it does not require specialist software, our results suggest that fitting the sine-wave model can provide more consistent estimates of the parasite growth rate. An additional advantage of fitting the sine-wave model is that it provides estimates of the parasite amplitude and life-cycle. However, fitting the sine-wave model requires at least six observations, and convergence of the model can be sensitive to starting values.

The Bayesian Information Criterion has been used as an indication of model fit, however mixed effects models with other nested random effects based on alternative model selection criteria result in similar parameter estimates and conclusions. Inclusion of the random effect for *m* in the QIMR-B studies had minimal or no improvement on the model fit. Therefore, the more parsimonious models with a nested random effect for a is presented.

A limitation of this report is the lack of consistency in processing parasitemia values. We used geometric mean of replicate parasitemia data for QIMR-B studies, whereas arithmetic mean was used in two studies from other research groups [14, 35], and other studies did not specify how parasitemia data was processed. This difference in parasitemia data processing may influence comparison between studies.

This report presents the parasite growth rate, PMR_48_, PMR_LC_, amplitude and life-cycle of *P. falciparum* 3D7 in a large number of subjects inoculated using the IBSM model under similar conditions. The parasite growth rates estimated using data from IBSM studies conducted by QIMR-B were comparable to estimates using data from other IBSM studies. The *P. falciparum* 3D7 parasite life-cycle estimated in this study can be used to calculate the PMR_LC_ in future VIS.

## NOTES

### Current affiliations

**Isabell Hoffman**. Paul-Ehrlich-Institut, Federal Institute for Vaccines and Biomedicines, 63225 Langen, Germany.

**Leesa F. Wockner.** BHP Group Limited, Brisbane, Australia.
