# Supplementary material for: Growth Rate of Plasmodium falciparum: Analysis of Parasite Growth Data from Malaria Volunteer Infection Studies
Source: J Infect Dis. 2019 Nov 4;221(6):963–72. doi: 10.1093/infdis/jiz557 (PMC7198127; doi:10.1093/infdis/jiz557)
Supplement: Supplementary file 1 [file JID-2019-INFDIS-JIZ-557-s1.docx]

**Growth Rate of *Plasmodium falciparum*: Analysis of Parasite Growth Data from Malaria Volunteer Infection Studies**

Leesa F. Wockner, Isabell Hoffmann, Lachlan Webb, Benjamin Mordmüller, Sean C. Murphy, James G. Kublin, Peter O’Rourke, James S. McCarthy, Louise Marquart

**SUPPLEMENTARY MATERIAL**

**METHODS**

**Parasite Growth Monitoring and Data Processing for Studies from Other Research Groups**

We estimated parasite growth parameters using data that were available to us from IBSM and sporozoite studies from other research groups. Parasitemia data obtained by PCR or reverse transcription PCR (RT-PCR) were processed as in the original publications. The PCR or RT-PCR methodologies used for each study are summarized in Table 2 of the manuscript. The data used for each analysis are presented in Supplementary Table 3 and summarized below.

**Duncan et al. (2011)** Parasitemia data were processed as per original publication [1]. Vaccine and control subjects were included in the analysis because the authors showed that vaccination did not affect PMR estimates. Samples obtained before the first detection of parasites were excluded from analysis. Non-detected parasitemia values occurring after the first positive recording were replaced with the lower limit of detection (LOD) of the qPCR assay, which was 20 parasites/mL.

**Bijker et al. (2013).** Only data from control groups were included in the analysis [2]. Data prior to the first positive parasitemia measurement were excluded from analysis. Subsequent non-detected parasitemia values were replaced with 10 parasites/mL. Data from subjects inoculated using the IBSM model or using a sporozoite model were modelled separately. Bijker-IBSM data were modeled from day 5 onwards because two subjects (Subject IDs 6 and 7 in original publication) first had positive parasitemia detected at day 2 and 3 respectively followed by non-detected values until day 5.

**Payne et al. (2016).** Parasitemia data were processed as per original publication [3]. Negative PCR results before first positive results were excluded from analysis (<20 parasites/mL). All subsequent values <20 parasites/mL or negative results were replaced with 10 parasites/mL.

**Sanderson et al. (2008).** Parasitemia data were processed as per original publication [4], which was the same procedure described for the Payne et al. (2016) study.

**Lawrence et al. (2000).** Replicate parasitemia data were available for this study [5]. Replicates were averaged on the log_10_ scale. Negative results were set to 1 parasite/mL. No other substitutions were implemented.

**Cheng et al. (1997).** Replicate parasitemia data were available for this study [6]. Replicates were averaged on the log_10_ scale. Negative results were set to 1 parasite/mL. No other substitutions were implemented. Subject 1 was excluded from analysis.

**Douglas et al. (2013).** Parasitemia data were processed as per original publication [7], which was the same procedure described for the Payne et al. (2016) study. The study by Sheehy et al. (2013) [8] presented in the original publication was excluded for analysis because we analyzed this study separately. We analyzed two datasets from the collection of studies presented in [7]: Douglas-SYBR Green, which used SYBR Green qPCR technology, and Douglas-TaqMan, which used TaqMan qPCR technology.

**Reuling et al. (2018).** We excluded from analysis intermittent positive parasitemia values reported in three subjects (Subjects IDs 2878, 3146, and 3259 in original publication [9]). For these three subjects, we included parasitemia values from the first two consecutive positive measurements (Subject 2878, from day 8; Subject 3146, from day 9.7; Subject 3259, from day 10.5). Data prior to the first parasitemia positive measurement were excluded from analysis. Subsequent negative results were replaced with 10 parasites/mL.

**Coffeng et al. (2017).** This report presented data from nine sporozoite studies in which subjects were infected by mosquito bites [10].. We excluded one subject from analysis (Subject ID ZONMW1.1270) as reported by Coffeng et al. (2017) [10]. Samples obtained before the first detection of parasites were excluded from analysis. All subsequent parasitemia values below the LOD (LOD = 20 parasites/mL for 38 individuals or LOD=200 parasites/mL for 18 individuals)or non-detected parasitemia values were replaced with LOD/2 of either 10 or 100 parasites/mL.

**Sheehy et al. (2013).** Parasitemia density data were processed as per original publication [8], which was the same procedure described for the Payne et al. (2016) study.

**Mordm**ü**ller et al. (2017), Sulyok et al.** (2017), **MALACHITE, PREMIVER.** Parasitemia data were processed as per original publication [11, 12]. All parasitemia values below the detection threshold (<6 parasites/mL) were replaced with 3 parasites/mL, except samples with values below the threshold level obtained before the first positive sample which were excluded. Only placebo subjects were made available for analysis.

**Murphy et al. (2018).** Parasitemia data were processed as per original publication [13]. Only data from placebo subjects were analyzed. Parasitemia values after the first positive sample and either not-detected or detected parasitemia values below the LOD (LOD = 20 parasites/mL) were replaced with 10 parasites/mL. Placebo subjects from Cohort 2b could not be used due to insufficient positive samples.

RESULTS

Sensitivity Analyses for Imputation Procedures for Non-Detected Parasitemia Values

Parameters were estimated separately for QIMR-B IBSM studies by log-linear and sine-wave mixed effects models for a range of imputation procedures for non-detected parasitemia values (ND). The distribution of low parasitemia estimates was 73 (6.5%) <8 parasites/mL, 104 (9.2%) <16 parasites/mL and 131 (11.6%) <32 parasites/mL, and 30 (2.9%), 50 (4.9%) and 65 (6.3%) respectively if only data from Day 5 onwards is considered. Combinations of parameter estimates from imputed values of 1, LOD/2 = 32, or missing for partial and full ND were compared and the results are presented in Supplementary Table 4. The combination of ND = 1 for each partial and full ND gave similar parameter estimates to those presented in the overall model for this study. However, imputation with ND = 32 or missing gave lower estimates. These results with a high proportion of low parasitemia values in the dataset confirm the substitution of a low value for ND. A further sensitivity analysis truncating all data prior to day 5 gave similar estimates of parasite growth rate for all imputation methods, indicating robustness across the proportion of censored data. For the imputation method presented in this study, the results from fitting the linear mixed model to data from Day 5 onwards has a similar parasite growth rate of 0.67 (95% CI: 0.63 – 0.71) compared to the 0.70 (95% CI: 0.67 – 0.73) as presented in the main analysis. Similarly, the results from fitting the sine-wave mixed model to data from Day 5 onwards has a similar parasite growth rate of 0.78 (95% CI: 0.76 – 0.81) compared to the 0.75 (95% CI: 0.73 – 0.77) as presented in the main analysis.

Imputation procedures and a range of analytical methods for appropriately including information from values below limit of quantification have been widely researched since the original studies of Beal [14] who proposed and evaluated seven methods. Senn et al [15], Marquart et al [16], and Boyer et al [17] further investigated analytical strategies where left censoring of qPCR data has been a common issue. There is scope for further research into hierarchical Markov Chain Monte Carlo (MCMC) methods, Tobit regression, and the use of mixed effects hierarchical models that use non-normal random effects to handle the censored data from parasitemia values estimated by nucleic acid tests.

Parasite Growth Rates of QIMR-B IBSM Studies Fitted by Subject and by Cohort

Parasite growth rate and parasite life-cycle of *P. falciparum* 3D7 estimated for QIMR-B IBSM studies fitting log-linear and sine-wave models were similar when data were fitted by subject, by cohort, and overall (Supplementary Table 5).

The sinusoidal parasite growth was not observed for one cohort (OZ439, Cohort 2). For this cohort, the life-cycle was fixed to the median life-cycle estimated overall to fit the sine-wave model by subject and by cohort. The median life-cycle estimated overall was 38.8 h (Supplementary Table 5).

The sine-wave growth model by subject did not converge for 41 of the 177 subjects because insufficient data were available to estimate all parameters (five data points or fewer). Parasite growth rates were estimated using a sine-wave model for the 136 subjects for whom the sine-wave model converged, and a log-linear model for the remaining 41 subjects. A two sample t-test indicated the parasite growth rates estimated using the log-linear model did not differ from the sine-wave model (difference in parasite growth rate = 0.01; 95% CI: -0.07–0.09; *P* = 0.88).

Variability was slightly higher for parasite growth rates estimated by subject than by cohort when data were fitted with log-linear or sine-wave models. Parasite growth rates for each cohort fitting a log-linear model are presented in Supplementary Table 6, and fitting a sine-wave model in Supplementary Table 7.

The mean parasite growth rates estimated using log-linear models were significantly different from parasite growth rates estimated using sine-wave models when the models were fitted by subject (*P* < 0.001) and by cohort (*P* = 0.007) (Supplementary Table 8). This difference was not significant when only subjects treated on Day 7 were included in the analysis by cohort.

**REFERENCES**

1. Duncan CJ, Sheehy SH, Ewer KJ, et al. Impact on malaria parasite multiplication rates in infected volunteers of the protein-in-adjuvant vaccine AMA1-C1/Alhydrogel+ CPG 7909. PLoS One **2011**; 6:e22271.

2. Bijker EM, Bastiaens GJ, Teirlinck AC, et al. Protection against malaria after immunization by chloroquine prophylaxis and sporozoites is mediated by preerythrocytic immunity. Proc Natl Acad Sci U S A **2013**; 110:7862-7.

3. Payne RO, Milne KH, Elias SC, et al. Demonstration of the blood-stage *Plasmodium falciparum* controlled human malaria infection model to assess efficacy of the *P. falciparum* apical membrane antigen 1 vaccine, FMP2.1/AS01. J Infect Dis **2016**; 213:1743-51.

4. Sanderson F, Andrews L, Douglas AD, Hunt-Cooke A, Bejon P, Hill AV. Blood-stage challenge for malaria vaccine efficacy trials: a pilot study with discussion of safety and potential value. Am J Trop Med Hyg **2008**; 78:878-83.

5. Lawrence G, Cheng Q, Reed C, et al. Effect of vaccination with 3 recombinant asexual-stage malaria antigens on initial growth rates of *Plasmodium falciparum* in non-immune volunteers. Vaccine **2000**; 18:1925-31.

6. Cheng Q, Lawrence G, Reed C, et al. Measurement of *Plasmodium falciparum* growth rates in vivo: a test of malaria vaccines. Am J Trop Med **1997**; 57:495-500.

7. Douglas AD, Edwards NJ, Duncan CJ, et al. Comparison of modeling methods to determine liver-to-blood inocula and parasite multiplication rates during controlled human malaria infection. J Infect Dis **2013**; 208:340-5.

8. Sheehy SH, Spencer AJ, Douglas AD, et al. Optimising controlled human malaria infection studies using cryopreserved *P. falciparum* parasites administered by needle and syringe. PLoS One **2013**; 8:e65960.

9. Reuling IJ, van de Schans LA, Coffeng LE, et al. A randomized feasibility trial comparing four antimalarial drug regimens to induce *Plasmodium falciparum* gametocytemia in the controlled human malaria infection model. Elife **2018**; 7.

10. Coffeng LE, Hermsen CC, Sauerwein RW, de Vlas SJ. The power of malaria vaccine trials using controlled human malaria infection. PLoS Comput Biol **2017**; 13:e1005255.

11. Mordmuller B, Surat G, Lagler H, et al. Sterile protection against human malaria by chemoattenuated PfSPZ vaccine. Nature **2017**; 542:445-9.

12. Sulyok M, Ruckle T, Roth A, et al. DSM265 for *Plasmodium falciparum* chemoprophylaxis: a randomised, double blinded, phase 1 trial with controlled human malaria infection. Lancet Infect Dis **2017**; 17:636-44.

13. Murphy SC, Duke ER, Shipman KJ, et al. A randomized trial evaluating the prophylactic activity of DSM265 against preerythrocytic *Plasmodium falciparum* infection during controlled human malarial infection by mosquito bites and direct venous inoculation. J Infect Dis **2018**; 217:693-702.

14. Beal SL. Ways to fit a PK model with some data below the quantification limit. J Pharmacokinet Pharmacodyn **2001**; 28:481-504.

15. Senn S, Holford N, Hockey H. The ghosts of departed quantities: approaches to dealing with observations below the limit of quantitation. Stat Med **2012**; 31:4280-95.

16. Marquart L, Baker M, O'Rourke P, McCarthy JS. Evaluating the pharmacodynamic effect of antimalarial drugs in clinical trials by quantitative PCR. Antimicrob Agents Chemother **2015**; 59:4249-59.

17. Boyer TC, Hanson T, Singer RS. Estimation of low quantity genes: a hierarchical model for analyzing censored quantitative real-time PCR data. PLoS One **2013**; 8:e64900.
