# Supplementary material for: Growth Rate of Plasmodium falciparum: Analysis of Parasite Growth Data from Malaria Volunteer Infection Studies
Source: J Infect Dis. 2019 Nov 4;221(6):963–72. doi: 10.1093/infdis/jiz557 (PMC7198127; doi:10.1093/infdis/jiz557)
Supplement: Supplementary file 2 [file JID-2019-INFDIS-JIZ-557-s2.docx]

**Supplementary Table 1. Details of QIMR-B IBSM Studies Analyzed in this Report**

| **Clinical Trial Name**  **[Reference]^a^** | **Clinical Trial ID** | **Cohort** | **Year^b^** | **No. of  Subjects** | **Treatment  Day^c^** | **Inoculum  Size^d^** | **Subject Gender** | | **Subject Age (Years)** | | |
| --- | --- | --- | --- | --- | --- | --- | --- | --- | --- | --- | --- |
|  |  |  |  |  |  |  | **Female** | **Male** | **18–24** | **25–29** | **≥30** |
| Mefloquine [20] | ACTRN12612000323820 | 1 | 2012 | 6 | 8 | 1800 | 2 | 4 | 3 | 2 | 1 |
|  |  | 2 | 2012 | 8 | 8 | 1800 | 5 | 3 | 6 | 2 | 0 |
|  |  | 3 | 2012 | 8 | 8, n=7 9, n=1 | 1800 | 4 | 4 | 1 | 5 | 2 |
| OZ439 [21] | ACTRN12612000814875 | 1 | 2012 | 8 | 8 | 1800 | 4 | 4 | 4 | 2 | 2 |
|  |  | 2 | 2012 | 8 | 8 | 1800 | 2 | 6 | 3 | 4 | 1 |
|  |  | 3 | 2012 | 8 | 7 | 1800 | 6 | 2 | 4 | 4 | 0 |
| DSM265 [22] | ACTRN12613000533796 | 1A | 2013 | 5 | 8 | 1800 | 0 | 5 | 3 | 2 | 0 |
|  |  | 1B | 2013 | 4 | 8 | 1800 | 0 | 4 | 3 | 0 | 1 |
| Piperaquine [23] | ACTRN12613000565741 | 1 | 2013 | 5 | 7 | 1800 | 0 | 5 | 4 | 1 | 0 |
|  |  | 2 | 2013 | 7 | 8 | 1800 | 1 | 6 | 4 | 2 | 1 |
|  |  | 3A | 2014 | 6 | 8 | 1800 | 4 | 2 | 5 | 1 | 0 |
|  |  | 3B | 2014 | 6 | 7 | 1800 | 4 | 2 | 4 | 1 | 1 |
| Ferroquine [24] | ACTRN12613001040752 | 1 | 2013 | 8 | 8 | 1800 | 5 | 3 | 4 | 3 | 1 |
| ACT-451840 [25] | ACTRN12614000781640 | 1 | 2014 | 8 | 7 | 1800 | 0 | 8 | 5 | 2 | 1 |
| MMV048 PIB [26] | NCT02230579 | 1 | 2014 | 6 | 7 | 1800 | 0 | 6 | 3 | 3 | 0 |
| OZ439/DSM265 [27] | NCT02389348 | 1 | 2015 | 8 | 7 | 1800 | 3 | 5 | 5 | 1 | 2 |
|  |  | 2 | 2015 | 5 | 7 | 1800 | 2 | 3 | 4 | 1 | 0 |
| EFITA/OZGAM^e^ [28] | NCT02431637/  NCT02431650 | 1/1 | 2015 | 6 | 7 | 2800 | 0 | 6 | 3 | 2 | 1 |
|  |  | 2/2 | 2016 | 4 | 8 | 2800 | 2 | 2 | 2 | 2 | 0 |
|  |  | 3/2B,3 | 2016 | 7 | 8 | 2800 | 2 | 5 | 3 | 2 | 2 |
| KAE609 [29] | NCT02543086 | 1 | 2015 | 8 | 7 | 1800 | 0 | 8 | 5 | 0 | 3 |
| DSMOZ-2 [30] | NCT02573857 | 1 | 2015 | 7 | 7 | 2800 | 2 | 5 | 4 | 2 | 1 |
| SJ733IBSMCS [31] | NCT02867059 | 1 | 2016 | 7 | 8 | 2800 | 0 | 7 | 4 | 1 | 2 |
|  |  | 2 | 2016 | 7 | 8 | 2300 | 0 | 7 | 2 | 3 | 2 |
|  |  | 2B | 2016 | 2 | 8 | 2300 | 0 | 2 | 2 | 0 | 0 |
| MMV048 Part B [32] | NCT02783833 | 1 | 2016 | 7 | 8 | 2800 | 0 | 7 | 3 | 1 | 3 |
|  |  | 2 | 2016 | 8 | 8 | 2800 | 0 | 8 | 3 | 1 | 4 |

^a^ References numbers as listed in the manuscript.

^b^Year when cohort started.

^c^Inoculation day was Day 0 for all cohorts.

^d^Approximate number of viable parasites.

^e^EFITA/OZGAM were run concurrently and therefore subjects in the same cohort received the same inoculum. These cohorts were analyzed as a single cohort. Cohort 2 of OZGAM was under-recruited and an additional cohort, Cohort 2B, was recruited to complete the cohort. OZGAM Cohort 2B was run concurrently with EFITA and OZGAM cohorts 3.

Abbreviation: QIMR-B, QIMR Berghofer.
