# Supplementary material for: Growth Rate of Plasmodium falciparum: Analysis of Parasite Growth Data from Malaria Volunteer Infection Studies
Source: J Infect Dis. 2019 Nov 4;221(6):963–72. doi: 10.1093/infdis/jiz557 (PMC7198127; doi:10.1093/infdis/jiz557)
Supplement: Supplementary file 4 [file JID-2019-INFDIS-JIZ-557-s4.docx]

**Supplementary Table 3. Data from Other IBSM and Sporozoite Studies Used for Analysis**

| **Study**  **[Reference]^a^** | **Handling of Non-Detected Parasitemia Values After First Positive Parasitemia Value** | **LOD or LLOQ in Original Publication** | **Data Source (Online or Author)** |
| --- | --- | --- | --- |
| Bijker et al. (2013) [34] | Non-detected parasitemia values after first positive value set to 10 parasites/mL | LOD = 20 parasites/mL | Author |
| Duncan et al. (2011) [35] | Non-detected parasitemia values after first positive value set to 20 parasites/mL | LOD = 20 parasites/mL | Available online |
| Payne et al. (2016) [14] | Non-detected parasitemia values after first positive value set to 10 parasites/mL | LLOQ = 20 parasites/mL,  LLOD = 5 parasites/mL | Available online |
| Sanderson et al. (2008) [15] | Non-detected parasitemia values after first positive value set to 10 parasites/mL | LLOQ = 20 parasites/mL | Author |
| Lawrence et al. (2000) [36] | No instances of all parasitemia replicates being non-detected values after first positive value | Not given | Author |
| Cheng et al. (1997) [37] | No instances of all parasitemia replicates being non-detected values after first positive value | Not given | Author |
| Reuling et al. (2018) [38] | Non-detected parasitemia values after first positive value set to 10 parasites/mL (as Bijker et al. 2013) | Assumed LOD = 20 parasites/mL as in Bijker et al. 2013 | Author |
| Douglas et al. (2013) [17] | Non-detected parasitemia values after first positive value set to 10 parasites/mL | LOD = 20 parasites/mL as in Bijker et al. 2013 | Available online |
| Coffeng et al. (2017) [19] | Non-detected parasitemia values after first positive value set to 10 parasites/mL | LOD = 20 parasites/mL | Available online |
| Sheehy et al. (2013) [8] | Non-detected parasitemia values after first positive value set to 10 parasites/mL | LLOQ = 20 parasites/mL,  LLOD = 5 parasites/mL | Available online |
| Mordmuller et al. (2017) [10] | Non-detected parasitemia values after first positive value set to 3 parasites/mL | Detection threshold =  6 parasites/mL  LOQ = 3 parasites/mL | Author |
| Sulyok et al. (2017) [40] | Non-detected parasitemia values after first positive value set to 3 parasites/mL | Detection threshold =  6 parasites/mL | Author |
| Murphy et al. (2018) [39] | Non-detected parasitemia values after first positive value set to 10 parasites/mL | LOD = 20 parasites/mL | Author |
| MALACHITE [41] | Non-detected parasitemia values after first positive value set to 3 parasites/mL | Detection threshold =  6 parasites/mL | Author |
| PREMIVER [42] | Non-detected parasitemia values after first positive value set to 3 parasites/mL | Detection threshold =  6 parasites/mL | Author |

^a^References numbers as listed in the manuscript.

Abbreviations: IBSM, induced blood stage malaria; LOD, limit of detection; LLOD, lower limit of detection; LLOQ: lower limit of quantification; ND, not detected.
