# Supplementary material for: Growth Rate of Plasmodium falciparum: Analysis of Parasite Growth Data from Malaria Volunteer Infection Studies
Source: J Infect Dis. 2019 Nov 4;221(6):963–72. doi: 10.1093/infdis/jiz557 (PMC7198127; doi:10.1093/infdis/jiz557)
Supplement: Supplementary file 5 [file JID-2019-INFDIS-JIZ-557-s5.docx]

**Supplementary Table 4. Sensitivity Analysis of QIMR-B Studies with Different Imputation Procedures for Non-Detected Parasitemia Values at and After the First Positive Parasitemia Value**

| **Analysis** |  | **Log-Linear Model** | **Sine-Wave model** | | |
| --- | --- | --- | --- | --- | --- |
| **Partial ND, n=105** | **Full ND Post Patency, n=26** | **Parasite Growth Rate per Day (SE) 95% CI** | **Parasite Growth Rate per Day (SE) 95% CI** | **Sine-Wave Amplitude (SE) 95% CI** | **Parasite Life Cycle in Hours (SE) 95% CI** |
| **Overall Model^a^** | | | | | |
| ND = 1 | ND = 64/2 | 0.71 (0.02) 0.67–0.74 | 0.75 (0.01) 0.73–0.77 | 0.63 (0.02) 0.59–0.66 | 38.8 (0.22) 38.3–39.2 |
| **Sensitivity Analyses** | | | | | |
| ND = 1 | ND = 1 | 0.71 (0.02) 0.67–0.75 | 0.77 (0.01) 0.75–0.80 | 0.71 (0.02) 0.67–0.76 | 38.4 (0.22) 38.0–38.9 |
| ND = 64/2 | ND = 64/2 | 0.63 (0.01) 0.60–0.65 | 0.68 (0.01) 0.66–0.70 | 0.57 (0.02) 0.54–0.60 | 37.8 (0.19) 37.5–38.2 |
| ND = missing value | ND = missing value | 0.62 (0.01) 0.59–0.64 | 0.67 (0.01) 0.66–0.69 | 0.56 (0.02) 0.53–0.59 | 37.8 (0.19) 37.4–38.1 |

^a^ Data from QIMR-B studies (studies from 2012 to 2017, n=177 subjects) were fitted overall using log-linear and sine-wave models.

Abbreviations: ND, not detected; QIMR-B, QIMR Berghofer; SE, standard error; CI: confidence interval.
