# Supplementary material for: Growth Rate of Plasmodium falciparum: Analysis of Parasite Growth Data from Malaria Volunteer Infection Studies
Source: J Infect Dis. 2019 Nov 4;221(6):963–72. doi: 10.1093/infdis/jiz557 (PMC7198127; doi:10.1093/infdis/jiz557)
Supplement: Supplementary file 6 [file JID-2019-INFDIS-JIZ-557-s6.docx]

**Supplementary Table 5. Parasite Growth Estimates for QIMR-B Studies Using Log-Linear and Sine-Wave Models by Subject, by Cohort and Overall**

|  | **Log-Linear Model** | **Sine-Wave Model^a^** | | |
| --- | --- | --- | --- | --- |
|  | **Parasite  Growth Rate per Day** | **Parasite  Growth Rate per Day** | **Sine-Wave  Amplitude** | **Parasite  Life-Cycle (h)** |
| **Parameters Estimated by Subject** |  |  |  |  |
| Mean (SD over subjects) | 0.72 (0.19) | 0.76 (0.16) | 0.70 (0.25) | 39.5 (4.04) |
| Min/max | 0.11/1.73 | 0.20/1.29 | 0.14/1.59 | 30.6/55.0 |
| Number of subjects | 177 | 136 | 136 | 128^b^ |
| **Parameters Estimated by Cohort** |  |  |  |  |
| Mean (SD over cohorts) | 0.73 (0.11) | 0.77 (0.10) | 0.68 (0.17) | 39.0 (2.46) |
| Min/max | 0.56/0.99 | 0.62/0.97 | 0.22/1.11 | 35.6/45.6 |
| Number of cohorts | 27 | 27 | 27 | 26^b^ |
| **Parameters Estimated Overall^c^** |  |  |  |  |
| Estimate  (95% CI) | 0.71  (0.67–0.74) | 0.75  (0.73–0.77) | 0.63  (0.59–0.66) | 38.8  (38.3–39.2) |

^a^The sine-wave model by subject did not converge for 41 of the 177 subjects because insufficient data were available.

^b^The parasite life-cycle was fixed to 38.4 hours (1.6 days) for Cohort 2 (n=8) of OZ439 study to enable fit.

^c^Modeling overall analyzed simultaneously data from the 177 subjects using mixed-effects models.

Abbreviations: QIMR-B, Queensland Institute of Medical Research Berghofer; CI, confidence interval; SD, standard deviation.
