# Supplementary material for: Growth Rate of Plasmodium falciparum: Analysis of Parasite Growth Data from Malaria Volunteer Infection Studies
Source: J Infect Dis. 2019 Nov 4;221(6):963–72. doi: 10.1093/infdis/jiz557 (PMC7198127; doi:10.1093/infdis/jiz557)
Supplement: Supplementary file 7 [file JID-2019-INFDIS-JIZ-557-s7.docx]

**Supplementary Table 6. Parasite Growth Parameters for QIMR-B IBSM Studies Using a Log-Linear Model Fitted by Cohort**

| **Clinical Trial Name**  **[Reference]^a^** | **Cohort No.** | **Y-intercept  (SE)** | **Parasite  Growth Rate (SE)** | **SD Random Effect for *a*^b^** |
| --- | --- | --- | --- | --- |
| Mefloquine [19] | 1 | 2.72 (0.09) | 0.57 (0.09) | <0.01 |
|  | 2 | 2.29 (0.12) | 0.56 (0.08) | 0.24 |
|  | 3 | 2.72 (0.15) | 0.76 (0.11) | 0.33 |
| OZ439 [20] | 1 | 3.05 (0.09) | 0.81 (0.12) | 0.11 |
|  | 2 | 3.03 (0.14) | 0.79 (0.06) | 0.38 |
|  | 3 | 2.57 (0.14) | 0.63 (0.14) | <0.01 |
| DSM265[21] | 1A | 2.56 (0.09) | 0.70 (0.08) | <0.01 |
|  | 1B | 2.92 (0.15) | 0.69 (0.10) | 0.21 |
| Piperaquine [22] | 1 | 2.59 (0.12) | 0.96 (0.13) | <0.01 |
|  | 2 | 2.34 (0.10) | 0.66 (0.07) | 0.16 |
|  | 3A | 2.76 (0.13) | 0.80 (0.07) | 0.22 |
|  | 3B | 2.29 (0.21) | 0.92 (0.15) | 0.40 |
| Ferroquine [23] | 1 | 2.84 (0.07) | 0.70 (0.06) | <0.01 |
| ACT-451840 [24] | 1 | 2.52 (0.15) | 0.80 (0.09) | 0.36 |
| MMV048 PIB [25] | 1 | 2.52 (0.11) | 0.79 (0.12) | <0.01 |
| OZ439/DSM265 [26] | 1 | 2.10 (0.11) | 0.71 (0.10) | 0.14 |
|  | 2 | 2.43 (0.11) | 0.99 (0.11) | <0.01 |
| EFITA/OZGAM [27] | 1/1 | 2.89 (0.12) | 0.71 (0.10) | 0.16 |
|  | 2/2 | 3.35 (0.08) | 0.73 (0.07) | <0.01 |
|  | 3/2B,3 | 3.17 (0.16) | 0.61 (0.06) | 0.37 |
| KAE609 [28] | 1 | 2.45 (0.09) | 0.75 (0.08) | <0.01 |
| DSMOZ-2 [29] | 1 | 2.52 (0.21) | 0.77 (0.10) | 0.49 |
| SJ733IBSMCS [30] | 1 | 3.01 (0.14) | 0.64 (0.06) | 0.30 |
|  | 2 | 2.51 (0.16) | 0.61 (0.06) | 0.36 |
|  | 2B | 2.86 (0.15) | 0.59 (0.15) | <0.01 |
| MMV048 Part B [31] | 1 | 3.21 (0.10) | 0.73 (0.05) | 0.21 |
|  | 2 | 2.83 (0.15) | 0.69 (0.07) | 0.37 |
| **Overall^c^** | **27** | **2.71 (0.06)** | **0.71 (0.02)** | **0.29/0.27^d^** |

^a^References numbers as listed in the manuscript.

^b^a = y-intercept.

^c^Estimates for the model fitted overall (analyzing data from all subjects simultaneously).

^d^Standard deviation of the random effect for a for cohort/subjects within a cohort.

Abbreviations: IBSM, induced blood stage malaria; QIMR-B, QIMR Berghofer; SE, standard error; SD, standard deviation.
