# Supplementary material for: Growth Rate of Plasmodium falciparum: Analysis of Parasite Growth Data from Malaria Volunteer Infection Studies
Source: J Infect Dis. 2019 Nov 4;221(6):963–72. doi: 10.1093/infdis/jiz557 (PMC7198127; doi:10.1093/infdis/jiz557)
Supplement: Supplementary file 8 [file JID-2019-INFDIS-JIZ-557-s8.docx]

**Supplementary Table 7. Parasite Growth Parameters for QIMR-B Studies Using a Sine-Wave Model Fitted by Cohort**

| **Clinical Trial Name [Reference]^a^** | **Cohort** | ***a*^b^ (SE)** | **Parasite  Growth Rate per Day  (SE)** | **Sine-Wave  Amplitude  (SE)** | **Sine-Wave Phase Shift (SE)** | **Parasite  Life-Cycle,  Days  (SE)** | **SD of Random Effect for *a*^b^** |
| --- | --- | --- | --- | --- | --- | --- | --- |
| Mefloquine [19] | 1 | 2.80 (0.07) | 0.68 (0.07) | 0.55 (0.08) | 4.57 (0.19) | 40.2 (2.23) | <0.01 |
|  | 2 | 2.20 (0.13) | 0.62 (0.06) | 0.73 (0.09) | 4.75 (0.11) | 45.6 (1.30) | 0.32 |
|  | 3 | 2.80 (0.16) | 0.83 (0.08) | 0.56 (0.08) | 4.37 (0.14) | 36.2 (1.57) | 0.40 |
| OZ439 [20] | 1 | 3.18 (0.10) | 0.82 (0.08) | 0.61 (0.07) | 4.19 (0.11) | 40.7 (2.17) | 0.23 |
|  | 2^c^ | 3.03 (0.15) | 0.83 (0.05) | 0.22 (0.05) | 3.27 (0.24) | 38.4 (NA)^c^ | 0.39 |
|  | 3 | 2.36 (0.11) | 0.69 (0.07) | 1.11 (0.09) | 4.39 (0.07) | 36.2 (0.58) | 0.25 |
| DSM265 [21] | 1A | 2.64 (0.08) | 0.80 (0.06) | 0.62 (0.11) | 2.94 (0.13) | 38.3 (1.02) | 0.10 |
|  | 1B | 2.95 (0.17) | 0.80 (0.04) | 0.64 (0.07) | 3.67 (0.11) | 39.3 (0.88) | 0.29 |
| Piperaquine [22] | 1 | 2.42 (0.11) | 0.96 (0.13) | 0.78 (0.17) | 3.64 (0.16) | 37.2 (1.68) | <0.01 |
|  | 2 | 2.32 (0.10) | 0.75 (0.06) | 0.51 (0.10) | 2.87 (0.17) | 37.5 (1.20) | 0.20 |
|  | 3A | 2.72 (0.13) | 0.90 (0.04) | 0.79 (0.06) | 5.91 (0.07) | 38.1 (0.47) | 0.30 |
|  | 3B | 2.11 (0.22) | 0.97 (0.11) | 0.90 (0.13) | 4.23 (0.11) | 36.4 (1.19) | 0.47 |
| Ferroquine [23] | 1 | 2.83 (0.08) | 0.79 (0.04) | 0.72 (0.07) | 5.61 (0.08) | 38.1 (0.56) | 0.17 |
| ACT-451840 [24] | 1 | 2.41 (0.17) | 0.70 (0.05) | 0.64 (0.07) | 3.87 (0.10) | 41.2 (1.45) | 0.43 |
| MMV048 PIB [25] | 1 | 2.30 (0.09) | 0.78 (0.05) | 0.98 (0.07) | 3.80 (0.06) | 37.8 (0.62) | 0.17 |
| OZ439/DSM265 [26] | 1 | 1.90 (0.11) | 0.80 (0.07) | 0.88 (0.09) | 3.77 (0.07) | 35.6 (0.71) | 0.26 |
|  | 2 | 2.25 (0.12) | 0.97 (0.08) | 0.81 (0.12) | 3.28 (0.11) | 38.2 (1.18) | 0.18 |
| EFITA/OZGAM [27] | 1/1 | 2.71 (0.12) | 0.70 (0.05) | 0.78 (0.08) | 3.49 (0.07) | 38.0 (0.81) | 0.26 |
|  | 2/2 | 3.36 (0.06) | 0.79 (0.04) | 0.56 (0.07) | 5.72 (0.11) | 40.0 (0.89) | 0.07 |
|  | 3/2B,3 | 3.16 (0.16) | 0.69 (0.04) | 0.63 (0.07) | 5.98 (0.10 | 38.9 (0.78) | 0.39 |
| KAE609 [28] | 1 | 2.41 (0.09) | 0.63 (0.06) | 0.70 (0.08) | 5.57 (0.10) | 37.6 (0.97) | 0.20 |
| DSMOZ-2 [29] | 1 | 2.46 (0.23) | 0.62 (0.07) | 0.66 (0.09) | 3.30 (0.14) | 43.2 (2.15) | 0.53 |
| SJ733IBSMCS [30] | 1 | 2.98 (0.15) | 0.73 (0.04) | 0.65 (0.07) | 5.83 (0.09) | 38.5 (0.64) | 0.36 |
|  | 2 | 2.48 (0.16) | 0.73 (0.05) | 0.58 (0.09) | 5.64 (0.13) | 37.9 (0.92) | 0.38 |
|  | 2B | 2.86 (0.13) | 0.76 (0.15) | 0.60 (0.21) | 3.16 (0.28) | 39.9 (2.98) | <0.01 |
| MMV048 Part B [31] | 1 | 3.29 (0.11) | 0.71 (0.04) | 0.47 (0.07) | 6.34 (0.14) | 45.2 (1.68) | 0.25 |
|  | 2 | 2.81 (0.16) | 0.78 (0.06) | 0.59 (0.10) | 5.96 (0.14) | 39.1 (1.05) | 0.41 |
| **Overall^d^** | **27** | **2.68 (0.06)** | **0.75 (0.01)** | **0.63 (0.02)** | **5.19 (0.12)** | **38.8 (0.22)** | **0.26/0.33^e^** |

^a^References numbers as listed in the manuscript.

^b^a = log10 parasitemia – (c × sin(k)).

^c^Period was fixed to 1.6 days for the model to converge.

^d^The SD of the random effect for the sine-wave phase shift by cohort was 0.61.

^e^Standard deviation of the random effect for a at the cohort level/subject within a cohort level.

Abbreviations: SE, standard error; SD, standard deviation; NA, not applicable.
