# Supplementary material for: Growth Rate of Plasmodium falciparum: Analysis of Parasite Growth Data from Malaria Volunteer Infection Studies
Source: J Infect Dis. 2019 Nov 4;221(6):963–72. doi: 10.1093/infdis/jiz557 (PMC7198127; doi:10.1093/infdis/jiz557)
Supplement: Supplementary file 9 [file JID-2019-INFDIS-JIZ-557-s9.docx]

**Supplementary Table 8. Comparison of QIMR-B Parasite Growth Rates Estimated Using Log-Linear or Sine-Wave Models by Subject and by Cohort**

|  | **Log-Linear Model**^a^ | **Sine-Wave Model**^a^ | **Difference (95% CI)** | ***P*-Value^b^** |
| --- | --- | --- | --- | --- |
| **Treatment at Day 7, 8 or 9** | |  |  |  |
| Subjects (n = 136^c^) | 0.72 | 0.76 | -0.045 (-0.064, -0.025) | <0.001 |
| Cohorts (n = 27) | 0.73 | 0.77 | -0.043 (-0.073, -0.012) | 0.007 |
| **Treatment at Day 7** |  |  |  |  |
| Subjects (n = 45) | 0.79 | 0.77 | 0.039 (0.003, 0.075) | 0.035 |
| Cohorts (n = 10) | 0.80 | 0.78 | 0.023 (-0.032, 0.078) | 0.38 |
| **Treatment at Day 8** |  |  |  |  |
| Subjects (n = 90) | 0.68 | 0.76 | -0.087 (-0.104, -0.070) | <0.001 |
| Cohorts (n = 16) | 0.68 | 0.76 | -0.081 (-0.105, -0.058) | <0.001 |

^a^Log-linear models fitted by subject used a linear regression, and by cohort a linear mixed-effects model. Sine-wave models fitted by subject used a non-linear regression, and by cohort a non-linear mixed-effects model.

^b^*P*-values were calculated using a paired t-test.

^c^Only 136 subjects were analyzed because sine-wave model did not fit for 41 subjects due to insufficient data points (only 5 data points were available).
