# Supplementary material for: Growth Rate of Plasmodium falciparum: Analysis of Parasite Growth Data from Malaria Volunteer Infection Studies
Source: J Infect Dis. 2019 Nov 4;221(6):963–72. doi: 10.1093/infdis/jiz557 (PMC7198127; doi:10.1093/infdis/jiz557)
Supplement: Supplementary file 10 [file JID-2019-INFDIS-JIZ-557-s10.docx]

**Supplementary Table 9. Parasite Growth Parameters for the QIMR-B Studies Estimated by Subject with Data Stratified by Gender, Age, and Inoculum Size**

| **Analysis** | **Log-Linear Model** | **Sine-Wave Model** | | |
| --- | --- | --- | --- | --- |
|  | **Parasite  Growth Rate per Day (95% CI)** | **Parasite  Growth Rate per Day  (95% CI)** | **Sine-Wave Amplitude  (95% CI)** | **Parasite Life-Cycle (h)**  **(95% CI)** |
| **Stratified Analysis by Gender** | | | | |
| Female (n=48) | 0.74 (0.67–0.81) | 0.81 (0.77–0.86) | 0.72 (0.65–0.78) | 38.4 (37.7–39.2) |
| Male (n=129) | 0.69 (0.66–0.73) | 0.73 (0.71–0.76) | 0.61 (0.57–0.65) | 38.8 (38.3–39.3) |
| Female vs Male  *P-*value^a^ | 0.10 | **<0.001** | **<0.001** | 0.29 |
| **Stratified Analysis by Age (years)** | | | | |
| 18–24 (n=96) | 0.70 (0.65–0.74) | 0.75 (0.72–0.78) | 0.63 (0.57–0.68) | 38.8 (38.2–39.4) |
| 25–29 (n=50) | 0.69 (0.63–0.75) | 0.76 (0.71–0.80) | 0.68 (0.62–0.74) | 39.0 (38.2–39.7) |
| ≥30 (n=31) | 0.73 (0.66–0.79) | 0.76 (0.72–0.81) | 0.62 (0.55–0.69) | 38.5 (37.7–39.4) |
| 18–24 vs 25–29  *P-*value^a^ | 0.82 | 0.83 | 0.22 | 0.75 |
| 18–24 vs ≥30  *P-*value^a^ | 0.52 | 0.74 | 0.93 | 0.64 |
| **Stratified Analysis by Inoculum Size (No. of Estimated Viable Parasites)^b^** | | | | |
| 1800 (n=122) | 0.72 (0.68–0.76) | 0.77 (0.74–0.80) | 0.66 (0.61–0.70) | 38.3 (37.8–38.9) |
| 2800 (n=46) | 0.69 (0.64–0.74) | 0.73 (0.70–0.77) | 0.56 (0.50–0.63) | 39.4 (38.6–40.2) |
| 1800 vs 2800  *P-*value^a^ | 0.44 | 0.22 | **0.025** | **0.033** |

^a^*P*-values were calculated using two-sample t-test using the estimated fixed effects and standard errors from the mixed effects models.

^b^Inoculum size of 2300 parasites was not included in the analysis because only 9 subjects were inoculated with this inoculum size.
